# EMT and Cancer Cell Stemness Associated With Chemotherapeutic Resistance in Esophageal Cancer

**DOI:** 10.3389/fonc.2021.672222

**Published:** 2021-06-03

**Authors:** Xiaojie Liu, Mingjing He, Linlin Li, Xiya Wang, Shuhua Han, Jinzhu Zhao, Yalong Dong, Mushtaq Ahmad, Leilei Li, Xueyan Zhang, Junfeng Huo, Yunfan Liu, Chengxue Pan, Cong Wang

**Affiliations:** ^1^ School of Pharmaceutical Sciences, Zhengzhou University, Zhengzhou, China; ^2^ State Key Laboratory of Esophageal Cancer Prevention and Treatment, Zhengzhou, China; ^3^ Key Laboratory of Advanced Drug Preparation Technologies, Ministry of Education of China, Zhengzhou, China

**Keywords:** esophageal cancer, drug resistance, paclitaxel, epithelial-mesenchymal transition, stemness

## Abstract

Drug resistance often occurs after chemotherapy in esophageal cancer patients, leading to cancer metastasis and recurrence. However, the relationship among cancer cell migration, recurrence and drug resistance in esophageal cancer drug-resistant cells has not been clearly explained. In this study, we constructed paclitaxel (PTX)-resistant esophageal cancer cells to explore the causes of drug resistance and poor prognosis after chemotherapy in esophageal cancer. Colony formation assay was used to evaluate the difference of colony formation between parental cells and drug resistance cells. Microsphere formation assay was used to examine the phenotype of stem cells. Wound healing and Transwell assays were used to detect the migration ability of drug-resistant cells. Western blotting and immunofluorescence assays were used to explore the mechanisms. Finally, we used nude mouse xenograft model to explore the tumor characteristics and the expression of relative proteins to verify our findings *in vivo*. Our study demonstrated that the cancer cell stemness characteristics exist in drug-resistant esophageal cancer cells, that expressed the biomarkers of stem cells and were prone to epithelial-mesenchymal transition (EMT). Our results suggested that the expression of EMT biomarkers and stemness-related proteins increased in esophageal cancer cells after continuously using chemotherapeutic drugs for a period of time. This study indicated that simultaneously targeting EMT and stemness could be a better strategy for the treatment of esophageal cancer drug resistance.

## Introduction

Esophageal cancer is one of the most common human digestive tract cancers and ranked as the sixth leading cause of cancer-related death worldwide (1). The 5-year overall survival rate of patients with advanced esophageal cancer is less than 10% (2). Esophageal squamous cell carcinoma (ESCC), as the predominant histologic type in China, seriously endangering people’s health (3). Surgical treatment, radiation therapy, chemotherapy, targeted therapy, and immunotherapy are accepted treatment options for patients. Among them, chemotherapy plays a dominant role. Chemotherapeutic agents, such as paclitaxel (PTX), are used widely for the treatment of advanced human cancers. However, long-term application of chemotherapeutic agents often leads to drug resistance even they are effective initially (4). In particular, tumor cells are more prone to invasion, metastasis, and recurrence after cancers develop chemotherapeutic resistance, leading to poor prognosis.

Cancer stem cells (CSCs) refer to a group of tumor cells with self-renewal capacity and differentiation potential, which can re-initiate tumor formation (5, 6). Studies have shown that the increase of recognizable stemness-related biomarkers in tumor cells is associated with driving the proliferation of tumor cells, the resistance of treatment and the recurrence of cancers (7–10). Previous studies have shown that CSCs exist in esophageal cancer (11). Although there is less consensus on biomarkers of CSCs in esophageal cancer so far, it has been determined that the poor prognosis of esophageal cancer patients is closely related to CSCs (12, 13).

CSCs also showed the tendency to invade and metastasize (14, 15). Epithelial-mesenchymal transition (EMT) plays a significant role in cancer metastasis and invasion (16). EMT, which transforms polarized epithelial cells to a motile mesenchymal phenotype, plays a primary role in morphologic changes in various physiological processes (17). A number of studies have shown that EMT contributes to the early spread of cancer cells, which is often activated during tumor invasion and metastasis (18). EMT is closely associated with poor prognosis in multiple types of cancers, including prostate cancer (19), breast cancer (20), lung cancer (21), hepatocellular carcinoma (22), and other cancers.

In this study, we investigate the association of cancer cell stemness along with EMT characteristics in drug-resistant esophageal cancer cells.

## Materials and Methods

### Materials

Paclitaxel was purchased from Hainan Quanxing Pharmaceutical Co., Ltd. (Hainan, China). MTT (3-(4,5-dimethyl-2-thiazolyl)-2,5-diphenyl-2-H-tetrazolium bromide) was purchased from Solarbio (Beijing, China). DMSO (Dimethyl sulfoxide) was acquired from Solarbio (Shanghai, China). Hoechst 33258 was purchased from Beyotime Biotechnology (Shanghai, China). Primary antibodies against ZO-1, Claudin-1, E-cadherin, β-catenin, Vimentin, N-cadherin, NANOG, SOX2, and OCT4 were purchased from Cell Signaling Technology, Inc. (Danvers, MA, USA).

### Cell Culture

Human esophageal cancer cell lines TE-1 was purchased from the Chinese Academy of Sciences (Shanghai, China), EC109 was purchased from the Institute of Basic Medical Sciences Chinese Academy of Medical Sciences & School of Basic Medicine Peking Union Medical College (Beijing, China). Esophageal cancer cell lines resistant to paclitaxel (TE-1/PTX and EC109/PTX) were established in our team according to the method of LIU-BIN GUO (23). All the cell lines were cultured in Roswell Park Memorial Institute (RPMI) 1640 (Biological Industries, Kibbutz Beit HaEmek, Israe) with 10% fetal bovine serum (FBS, purchased from Biological Industries, Kibbutz Beit HaEmek, Israel). Cells were incubated at 37°C in a humidified atmosphere containing 5% CO_2_.

### Cell Sensitivity Assay

Cell sensitivity to PTX was examined by MTT assay. Briefly, cells were seeded in the 96-well plate at a density of 4.5 × 10^3^ cells per well and incubated overnight. After that, the medium was changed to fresh medium containing various concentrations of PTX. After 72 h, 20 μl MTT was added to each well and cells were incubated for 4 h with MTT at the concentration of 500 mg/ml. Then, the precipitate was dissolved completely by 150 μl DMSO and the light absorbance was measured at 570 nm using the Multiskan Spectrum spectrophotometer (BioTek Instruments, Inc. Vermont, USA).

### Wound Healing Assay and Transwell Assay

Cells were seeded in six-well plate overnight and a sterile 100 μl pipette tip was used to scraped off a part of cells in each well. After that, plates were washed twice with PBS buffer solution to remove the detached cells. Subsequently, cells were incubated in culture medium with 2% FBS. The wound gaps were acquired by a microscope connected to a digital camera. After 24 and 48 h incubation, the wound gaps were acquired again, and the migration rates were evaluated.

Transwell assays were performed by Transwell chamber (Corning Life Sciences, NY, USA) in a 24-well plate. About 1 × 10^4^ EC109 and EC109/PTX cells and 1 × 10^3^ TE-1 and TE-1/PTX cells were added in the upper chamber in 300 μl culture medium with 0% FBS and 600 μl culture medium with 20% FBS was placed in the lower chamber. The cells were incubated at 37°C with 5% CO_2_ for 24 h. At the end, the cells were stained by 1% crystal violet solution and imaged under a Nikon microscope.

### Colony Formation Assay

1 × 10^3^ cells were seeded per well in 6-wells plate with culture medium with 10% FBS. The cells were incubated for 10 days. After that, the cells were washed three times with PBS and fixed in methanol for 30 min. Cells were stained with 1% crystal violet for 30 min. Then, the cells were washed with PBS at least five times.

### Sphere Formation Assay

200 cells of EC109, EC109/PTX, and TE-1, TE-1/PTX were cultured in serum-free medium DMEM/F12 containing supplement 2% B27 (Gibco, USA) and 0.1% growth factor EGF (Gibco, USA) in the ultra-low adsorption 96-well plate. Take photos every 24 h for a week under the microscope. The sphere numbers (diameter ≥50 μm) were calculated.

### Limit Dilution Assay -Sphere Formation Assay

Limit dilution assay (LDA) was used to select the tight cloning of cells. 30 to 50 cells were seeded in the 6-well plates. After 2 to 3 weeks, the cells of tight cloning were selected under microscope. Cells were suspended, counted, and cultured in the medium of cancer stem cells (98 ml DMEM/F12 + 2 μg EGF + 2 μg bFGF + 275 2 μl insulin + 2 ml B27) in the ultra-low adsorption 24-well plate. 10 days later, take photos under the microscope.

### Western Blotting

Cells were lysed on ice by RIPA lysis buffer containing phosphatase inhibitor cocktail and 1% protease for 30 min. Then, the lysates of the cells were centrifuged at 12,000 rpm for 10 min at 4°C and supernatant liquid were collected. The lysates were measured for protein concentration with a BCA protein assay kit. Total protein was resolved in 10% polyacrylamide gel by SDS-PAGE and transferred to polyvinylidene difluoride membranes. The nonspecific binding was blocked by 5% non-fat milk. The membranes were incubated with primary antibodies overnight at 4°C and then incubated with the secondary antibodies for 2 h at room temperature. The resolved protein bands were visualized by enhanced chemiluminescence (ECL) detection system. The densitometric analysis were performed using Image J software.

### Immunofluorescence Analysis

The cells were seeded in 24-well plates. After incubating overnight, the cells were fixed with methanol, permeabilized 10 min with 0.1% Triton X-100, blocked 1 h with 5% BSA and incubated with the primary antibodies (SOX2 1: 200, NANOG 1: 200, OCT4 1: 200) at 4°C overnight. After that, the cells were washed with PBS and incubated 1 h with Alexa Fluor 561 goat anti-rabbit IgG and Alexa Fluor 488 goat anti- rabbit IgG at room temperature. The nuclei of the cells were counterstained 20 min with 10 μg/ml of Hoechst 33258 in the dark. Images were acquired by laser scanning confocal microscopy (Nikon, A1, Tokyo, Japan). Target protein staining was presented in red (TE-1 and TE-1/PTX) and green (EC109 and EC109/PTX), and the nuclei were stained with Hoechst 33258 (blue).

### Tumor Growth *In Vivo*

Animal experiments were approved by the Institutional Animal Care and Use Committee of the Zhengzhou University. Approximately 5-week-old female nude mice were purchased from Hunan SJA Animal Laboratory Co. Ltd. The xenograft tumor model of nude mice was established. Briefly, 3 × 10^6^ EC109 cells and EC109/PTX cells were subcutaneously implanted into the right scapula of the mice. The volume of the tumors and the weight of the mice were measured every 2 days. Tumor size were calculated using the formula (A×B×B)/2 (A was the longest diameter and B was the shortest diameter of the tumor). The mice were sacrificed after 20 days. Then, tumors were dissected and weighed. Besides, the whole blood and the serum were obtained before the mice were sacrificed.

### Hematoxylin-Eosin Staining

Tumor tissues were embedded in paraffin, and the paraffin-embedded tissue was cut into 4- to 5-μm slices with a microtome. The slices were pasted on clean slides with ultrapure water, heated slightly with an alcohol lamp to make them flat. After that the paraffin slices were soaked in xylene for 20 min, they were soaked in gradient alcohol, soaked in ultrapure water for 5 min, then, soaked in PBS 20 min twice to carry out dewaxing and hydration. The slices were stained in hematoxylin staining solution for 2 min, rinsed with tap water and put into 1% alcoholic hydrochloric acid to decolorize and 1% ammonia solution to turn blue, and the nuclear staining was observed under a microscope, followed by putting into eosin staining solution for 1 min, tap water rinse and the staining was observed under the microscope. Before covering the slices, slides were dehydrated in gradient alcohol and cleared in xylene. They were sealed with neutral resin. The slices were dried and photographed using the Leica DM 3000 microscope at 200× magnification.

### Statistical Analysis

All the experiments were independently repeated three times. Data were shown as mean ± SD. Statistical analysis was performed using one-way ANOVA, using the GraphPad Prism 8.0. All comparisons were made relative to controls and significance of difference was indicated as * *P* < 0.05 and **P < 0.01.

## Results

### Characterization of the Parental Cells and the Drug-Resistant Cells

**Figure 1:**
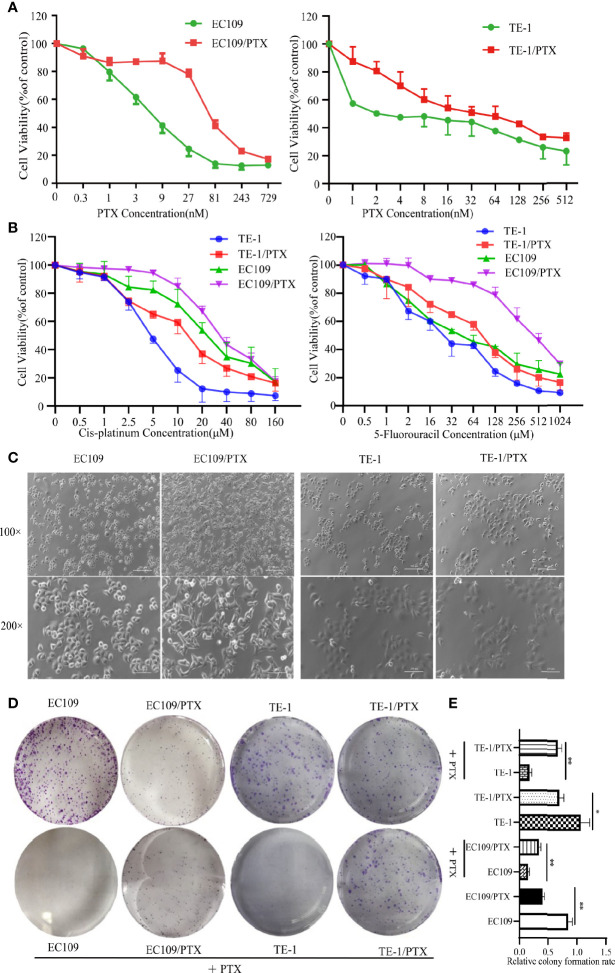
PTX-resistant EC109 and TE-1cells. **(A, B)** MTT assay on the survival of parental cells and PTX-resistant cells under the treatment with PTX, Cis-platinum and 5-Fluorouracil. **(C)** Cell morphology photographed under an inverted microscope. Scale bar: 500 µm (100×), 250 µm (200×). **(D)** Colony formation assay on the two groups of cells and the corresponding PTX solvent as control. **(E)** The corresponding statistical results of colony formation. *P < 0.05 indicates statistically significance *vs*. parental cells. **P < 0.01 indicates highly statistically significant *vs*. parental cells.

EC109 and TE-1 were treated with different concentrations of PTX for 72 h, and MTT assay was applied to evaluate the sensitivity of cells to PTX. The results showed that the IC_50_ value is about 8.69 nmol and 93.88 nmol in the EC109 and its drug-resistant cells (EC109/PTX), respectively. The PTX-resistant index is 10.80. The IC_50_ value is about 5.47 nmol and 56.84 nmol in the TE-1 and its drug-resistant cells (TE-1/PTX), respectively. The PTX-resistant index is 10.38 **(** **Figure 1A** and **Table 1** **)**. This result indicated that EC109/PTX and TE-1/PTX exhibited moderate drug resistance. Besides, we applied Cis-platinum and 5-Fluorouracil as the other drug sensitivity results upon PTX-resistant esophageal cancer cells. As a result, the Cis-platinum-resistant index is 2.36 (TE-1/PTX) and 1.54 (EC109/PTX), showed as mild drug resistance. The 5-Fluorouracil-resistant index is 3.10 (TE-1/PTX) and 9.93 (EC109/PTX), showed as mild drug resistance (TE-1/PTX) and moderate drug resistance (EC109/PTX). In other words, the resistant cells we stimulated were not only more resistant to the inducible drug PTX than the other therapeutic drugs but also showed different degrees of resistance to other therapeutic drugs **(** **Figure 1B** and **Table 2** **)**. Morphologically, EC109 and TE-1 had regular form and uniform size, in contrast, EC109/PTX and TE-1/PTX lost their original shape and became elongated **(** **Figure 1C** **)**, indicating that the cells changed after they gained drug resistance. The colony formation ability of the parental cells was significantly higher than that of the drug-resistant cancer cells in the colony formation assay. While when the parental cells and resistant cells were treated with PTX solvent as control, the colony formation ability of the parental cells was significantly lower than that of the drug-resistant cancer cells. This result further suggested that the drug-resistant cells were more resistant to paclitaxel than the parental cells **(** **Figures 1D, E** **)**.

**Table 1:** Drug sensitivity tests (PTX).

Cell lines	IC_50_ ± SD (nM)	Resistant Index
**EC109**	8.69 ± 2.06	–
**EC109/PTX**	93.88 ± 7.36	10.80 ± 2.32
**TE-1**	5.47 ± 0.18	–
**TE-1/PTX**	56.84 ± 2.31	10.38 ± 0.08

The results are shown as mean ± SD.

**Table 2:** Drug sensitivity tests (Cis-platinum and 5-Fluorouracil).

Cell lines	IC_50_ ± SD (Cis-platinum, μM)	Resistant Index	IC_50_ ± SD (5-Fluorouracil, μM)	Resistant Index
**EC109**	5.52 ± 0.73	–	21.53 ± 4.04	–
**EC109/PTX**	13.01 ± 0.14	2.36 ± 0.03	66.81 ± 2.18	3.10 ± 1.29
**TE-1**	25.84 ± 1.45	–	47.29 ± 3.67	–
**TE-1/PTX**	39.72 ± 3.09	1.54 ± 0.29	469.64 ± 24.31	9.93 ± 0.49

The results are shown as mean ± SD.

### PTX-Treated Cancer Cell Lines Showed Properties of Cancer Cell Stemness

**Figure 2:**
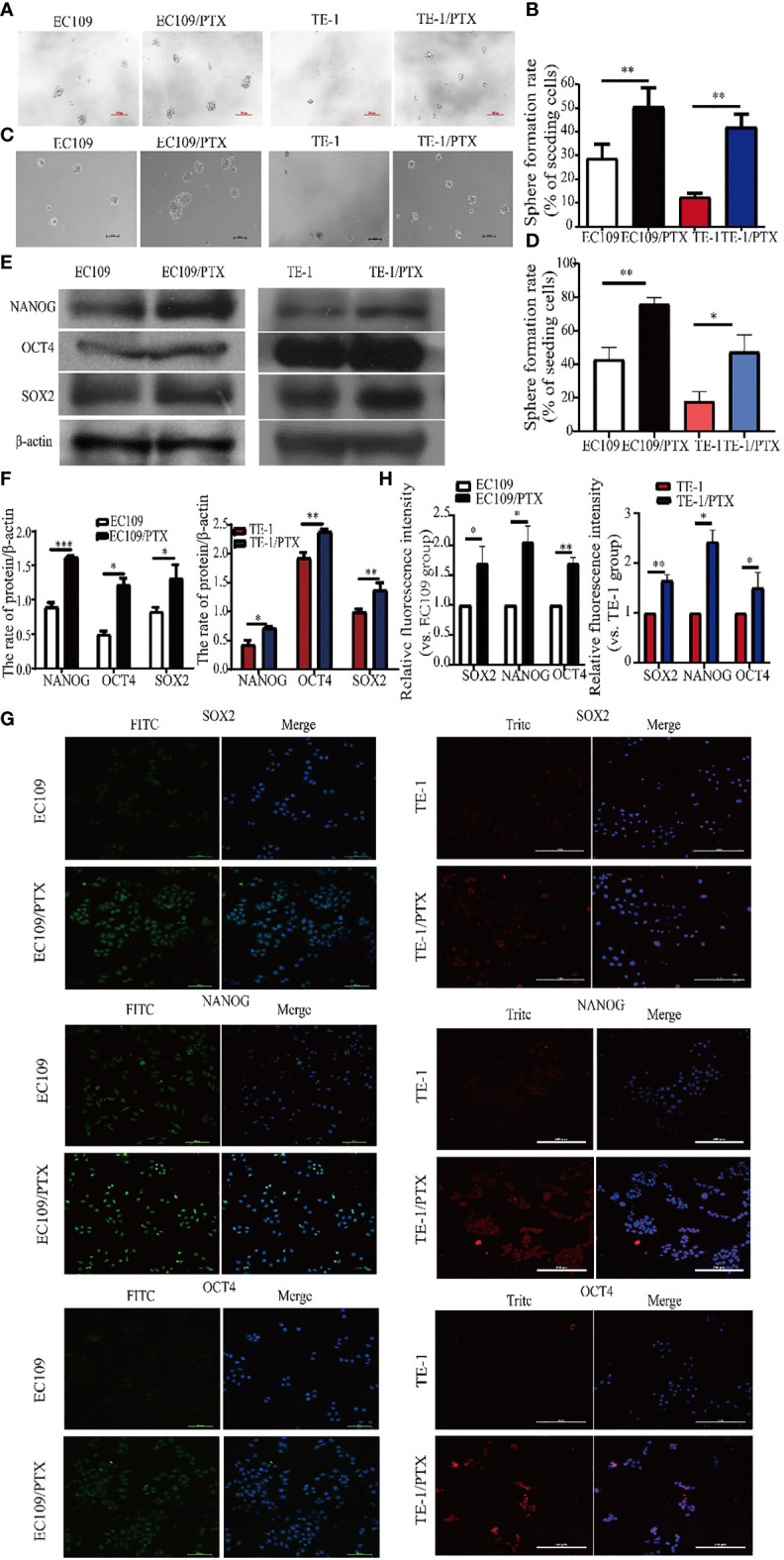
Enhanced cancer cell stemness in the drug-resistant cells. **(A, B)** Sphere formation assay on the two groups of cells and the corresponding statistical results. Scale bar: 100 µm. **(C, D)** LDA-Sphere formation assay on the two groups of cells and the corresponding statistical results. Scale bar: 100 µm. **(E, F)** Western blotting on the stem cell protein biomarkers and the corresponding statistical results. **(G, H)** Immunofluorescence analysis detected the expression of stem cell protein biomarkers NANOG, OCT4 and SOX2 in the two groups of cells. Scale bar: 100 µm (TE-1, TE-1/PTX), 200 µm (EC109, EC109/PTX). *P < 0.05 indicates statistically significance *vs*. parental cells. **P < 0.01 indicates highly statistically significant *vs*. parental cells.

The microsphere formation assay was used to test the property of cancer cell stemness, and the results showed that the microsphere formation rate in EC109/PTX and TE-1/PTX is 1.63-fold and 3.35-fold greater than their parental cells respectively **(** **Figures 2A, B** **)**. Besides, we used LDA to repeat sphere formation. The results showed that the microsphere formation rate in EC109/PTX and TE-1/PTX was greater than their parental cells respectively. Also, in general, the microsphere in EC109/PTX and TE-1/PTX was bigger than that in their parental cells, respectively **(** **Figures 2C, D** **)**. These results indicated that the self-renewal ability of drug-resistant cells is stronger than the parental cells. Western blotting assay was used to examine the expression of related proteins, and the result showed that the expression of embryonic stem cell transcription factors NANOG, OCT4, and SOX2 enhanced in the drug-resistant cell lines (TE-1/PTX and EC109/PTX) **(** **Figures 2E, F** **)**. The immunofluorescence analysis showed similar results as the Western blotting **(** **Figures 2G, H** **)**.

### The Migratory Ability Enhanced and the Expression of EMT-Related Protein Changed in the drug-Resistant Cells

**Figure 3:**
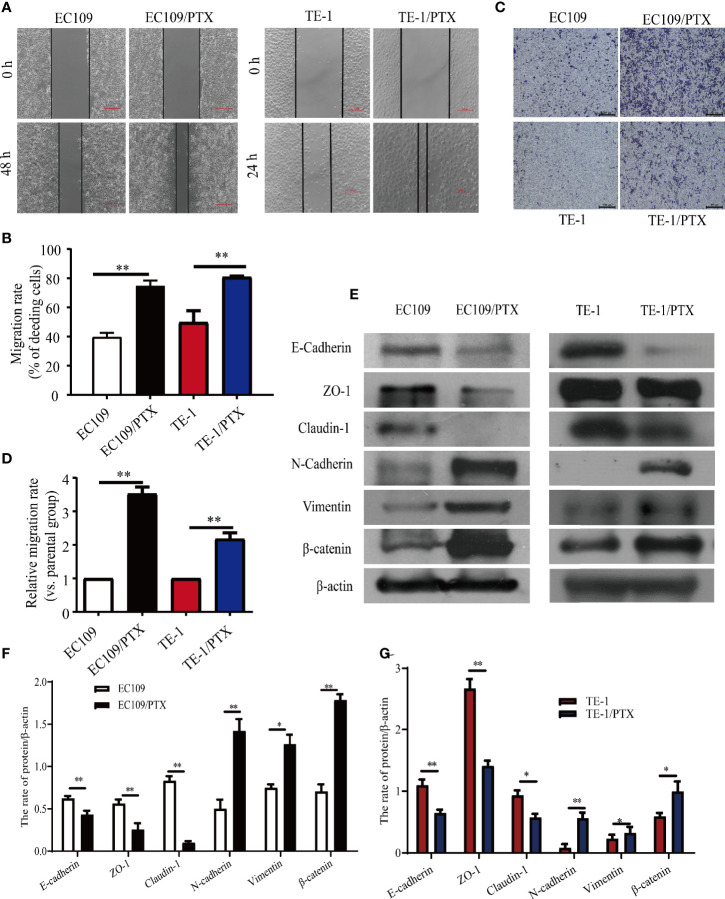
The migratory ability enhanced and the expression of EMT biomarkers changed in the PTX-treated cells. **(A, B)** Wound healing assay was used for the migration ability of the two groups of the cells and the corresponding statistical results. Scale bar: 500 µm. **(C, D)** Transwell assay on the two groups of the cells and the corresponding statistical results. Scale bar: 500 µm. **(E–G)** Western blotting evaluated the representative proteins of EMT and the corresponding statistical results. *P < 0.05 indicates statistically significance *vs*. parental cells. **P < 0.01 indicates highly statistically significant *vs*. parental cells.

Wound healing and Transwell assays were used to investigate the difference of cell migration ability in the drug sensitive and -resistant cells. The results of the wound healing assay indicated that after culturing the cells for 24 and 48 h, the size of the healed space in EC109 and TE-1 cells is 1.875-fold and 1.64-fold greater than that of EC109/PTX and TE-1/PTX cells **(** **Figures 3A, B** **)**. The results of the Transwell assay indicated that the number of the migrated EC109/PTX and TE-1/PTX cells was 4.2-fold and 2.3-fold of that of EC109 and TE-1 cells **(** **Figures 3C, D** **)**. These results suggested that once the cells gained the drug-resistant property, they have stronger ability of migration and invasion than their parental cells. Western blotting assay was used to explore the expression of EMT-related proteins. The results showed that the expression of the epithelial protein biomarkers Claudin-1, ZO-1, and E-cadherin were decreased in the drug-resistant cells, while the expression of the mesenchymal protein biomarkers Vimentin, N-cadherin, and the transcription factor β-catenin were increased **(** **Figures 3E–G** **)**.

### EMT-Related and Stemness-Related Proteins in the Drug-Resistant Cells Increased *In Vivo*

**Figure 4:**
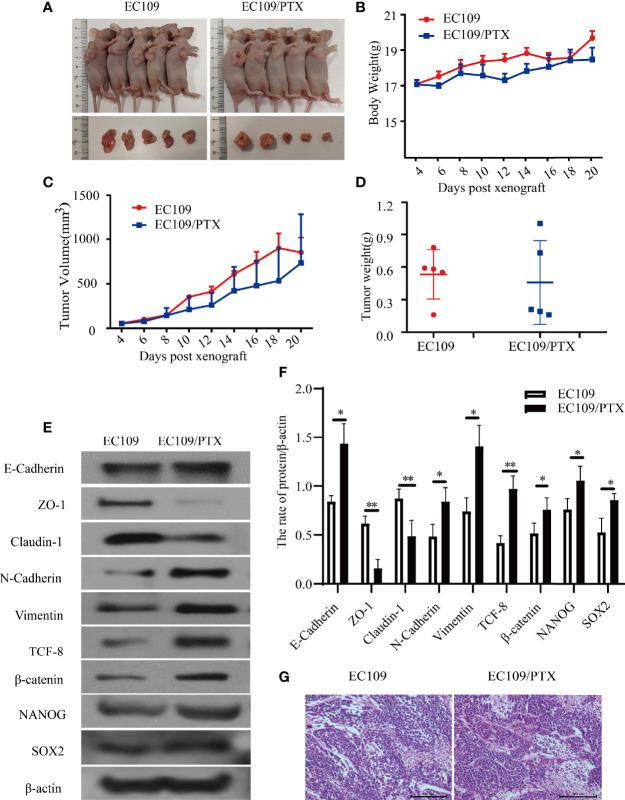
EC109 and EC109/PTX cell growth *in vivo* and the expression of EMT-related and stemness-related proteins. **(A)** The mice and tumor tissues of the two groups after implanting tumor cells for 20 days. **(B–D)** The body weight, tumor volume and tumor weight of the mice and tumor. **(E, F)** Western blotting for the expression of the EMT-related and stemness-related proteins and the corresponding statistical results. **(G)** Tumor tissue was stained with HE, the nucleus was stained blue-violet, and the cytoplasm was stained red. Scale bar: 200 µm. *P < 0.05 indicates statistically significance *vs*. EC109 cells. **P < 0.01 indicates highly statistically significant *vs*. EC109 cells.

To further explore the EMT and stemness in the drug-resistant esophageal cancer, we applied the xenograft tumor model in nude mice. It was found that, there is hardly any difference in the Blood Biochemical Index and Serum Biochemical Indices(ALT, AST and creatinine), except the uric acid and white blood cells. The level of Uric acid in the drug-resistant cells group is lower than that in the parental cells group, but both of them are in the normal range. It was also found that there was no significant difference in liver and kidney function between the mice in the parental cells group and the drug-resistant cells group. The difference in the white blood cells exist in the PTX -resistant cells group, and might due to the bone marrow inhibition, causing decrease of white blood cells **(** **Tables 3**, **4** **)**. The mice were sacrificed when we finished collecting blood from the eyeball. The average volume and the tumor weight of the in the mice of implanting EC109/PTX were smaller than the parental cells-group while the body weight was minimally affected **(** **Figures 4A–D** **)**. The expression of EMT- and stemness-related proteins was analyzed. It was found that except E-cadherin, the expression of epithelial protein biomarkers ZO-1 and Claudin-1 were decreased in the group of EC109/PTX cells, while the expression of mesenchymal protein biomarkers Vimentin, N-cadherin, and the transcription factor TCF-8 and β-catenin were increased. The expression stemness-related proteins SOX2 and NANOG enhanced **(** **Figures 4E, F** **)**. Besides, E-cadherin is related to proliferation closely and the volume of tumor tissue in the parent group increased significantly compared with the drug-resistant group *in vivo*. The expression of E-cadherin enhanced *in vivo*, which may be caused by the xenograft tumor model. In addition, hematoxylin-eosin staining was performed in tumor tissues **(** **Figure 4G** **)**. These *in vivo* results showed that once the tumor cells became drug resistant, the expression of the CSCs biomarkers and the EMT biomarkers enhanced. All the results were in consistent with the results *in vitro*.

**Table 3:** Biochemical Indexes of Serum.

Biochemical Indexes	EC109	EC109/PTX
**ALT**	13.41 ± 1.89 IU/L	15.01 ± 1.18 IU/L
**AST**	28.04 ± 6.39 IU/L	29.75 ± 1.93 IU/L
**Creatinine**	15.63 ± 2.82 mol/L	20.40 ± 3.21 mol/L
**Uric acid**	107.50 ± 12.24 mol/L	74.28 ± 16.14 mol/L **

**P < 0.01 indicates highly statistically significant vs. EC109 cells.

**Table 4:** Blood Biochemical Index.

Blood Biochemical Index	EC109	EC109/PTX
**WBC**	7.49 ± 1.80 10^9^/L	3.81 ± 2.20 10^9^/L **
**RBC**	9.81 ± 0.39 10^12^/L	8.95 ± 0.71 10^12^/L
**HGB**	155.83 ± 5.46 g/L	146.60 ± 5.31 g/L
**HCT**	49.7 ± 2.19%	45.23 ± 2.98%
**MCV**	50.62 ± 0.57 fL	50.38 ± 0.80 fL
**MCH**	15.85 ± 0.28 pg	16.50 ± 1.52 pg
**MCHC**	313.5 ± 3.86 g/L	327.40 ± 25.41 g/L
**RDW-S**	14.13 ± 0.26%	14.33 ± 0.59%
**RDW-SD**	29.67 ± 0.75 FI	30.00 ± 1.55 FI
**PLT**	826.67 ± 80.77 10^9^/L	727.60 ± 115.32 10^9^/L
**PCT**	0.59 ± 0.04%	0.52 ± 0.10%
**MPV**	7.13 ± 0.35 fL	7.06 ± 0.32 fL
**PDW**	16.63 ± 0.11%	15.94 ± 0.28%

**P < 0.01 indicates highly statistically significant vs. EC109 cells.

## Discussion

**Figure 5:**
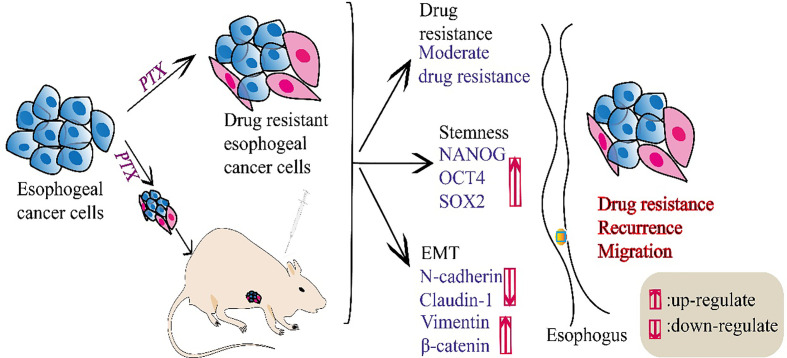
A schema summarized this study.

Chemotherapy using PTX as a first-line chemotherapeutic agent is the main treatment for patients with esophageal cancer (24, 25). However, drug resistance occurs in a large proportion of patients after treatment, which affects the efficacy of PTX treatment (26). In this study, we showed that esophageal cancer cells became less sensitive to PTX and exhibited moderate-drug-resistance after treatment with PTX. Moreover, the PTX-resistant cells develop stemness characteristics. We found that the expression of stem cell biomarkers NANOG, OCT4, SOX2 increased in the PTX-resistant esophageal cancer cells. Simultaneously, we found that the expression of epithelial biomarkers decreased and the mesenchymal biomarkers increased in drug-resistant esophageal cancer cells. Also, we verified these findings *in vivo* (**Figure 5**). OCT4, SOX2 and NANOG, as the embryonic stem cell transcription factors, transform cancer cells to stem-like cell phenotype in different tumor types (27). Among them, NANOG is an important transcription factor involved in the regulation of cell stemness (28). NANOG plays an important role in self-renewal, undifferentiated state and differentiation ability, high tumorigenicity, and resistance to current standard chemotherapy and radiotherapy (29–31). It can be activated by different transcription factors, such as OCT4 and SOX2 (32, 33). Previous studies have shown that NANOG is closely related to the poor prognosis of cancers (34).

In addition, we also found that after the cells were induced into drug-resistant esophageal cancer cells by PTX, the expression of EMT-related transcription factor β-catenin was up-regulated, the expression of epithelial biomarkers Claudin-1, ZO-1 and E-cadherin was reduced and the mesenchymal biomarkers N-cadherin, Vimentin expression increased. EMT plays an important role in the infiltration and metastasis of tumor cells, the formation of tumor drug resistance and tumor stem cells (35). The results indicated that once esophageal cancer cells become drug resistant, the expression of both cancer stem cell biomarkers and EMT-related biomarkers changed, which indicated clinical recurrence and migration ability (36, 37). Some studies have demonstrated that mesenchymal cells share similar molecular characteristics with CSCs, to some extent (38). Previous studies also revealed that the activation of EMT program led to the acquisition of the characteristics of CSCs in tumor cells (39).

In conclusion, our study suggested that drug resistance and cancer cell stemness develop at the same time during chemotherapy in esophageal cancer. The emergence of stemness explained why recurrence and metastasis occurred after drug resistance and caused poor prognosis. Our results indicated that targeting EMT and stemness at the same time in drug-resistant esophageal cancer could provide a better therapeutic effect.

## Data Availability Statement

The original contributions presented in the study are included in the article/supplementary material. Further inquiries can be directed to the corresponding authors.

## Ethics Statement

The animal study was reviewed and approved by the Institutional Animal Care and Use Committee of the Zhengzhou University.

## Author Contributions

CW, CP, and XL designed and performed the experiments. XL drafted the manuscript. MH, LinL, XW, SH, JZ, YD, MA, LeiL, XZ, JH, and YL participated in the experiments and revised the manuscript. CW supervised this study. All authors contributed to the article and approved the submitted version.

## Funding

This work was supported by grants from the National Natural Science Foundation of China (no. U1904156) and Zhengzhou University Student Innovation Experiment Project (UIEP).

## Conflict of Interest

The authors declare that the research was conducted in the absence of any commercial or financial relationships that could be construed as a potential conflict of interest.
